# Atypical Presentation of a Patient With Progressive Multifocal Leukoencephalopathy

**DOI:** 10.7759/cureus.62545

**Published:** 2024-06-17

**Authors:** Saimounika Adapa, Nilesh Jagdale, Kalyan K Vutukuru, Mohith Prakash Kondapalli, Sonali Agarwal

**Affiliations:** 1 Department of General Medicine, Dr. D. Y. Patil Medical College, Hospital and Research Centre, Pune, IND

**Keywords:** antiretroviral therapy, john cunningham (jc) virus, immunocompromised, pml, progressive multifocal leukoencephalopathy (pml), hiv aids, demyelination

## Abstract

Progressive multifocal leukoencephalopathy (PML) is a rare, demyelinating infectious disease of the central nervous system, primarily affecting immunosuppressed individuals, such as those with acquired immunodeficiency syndrome (AIDS) or undergoing immunosuppressive therapy. The causative agent is the dormant John Cunningham (JC) polyomavirus, which reactivates in immunocompromised patients. PML is diagnosed through clinical observations, imaging, and polymerase chain reaction (PCR) analysis, detecting JC virus deoxyribonucleic acid (DNA) in the cerebrospinal fluid (CSF). Here, we report a case of a 42-year-old male, recently diagnosed with human immunodeficiency virus (HIV), who presented with slurred speech, difficulty articulating, tingling in both feet, difficulty walking, and significant weight loss. Examination revealed absent reflexes, coordination impairment, and diminished vibration sense. Blood tests showed anemia, elevated D-dimer, and HIV-1 positivity with a low CD4 count. CSF analysis indicated a lymphocytic profile with elevated protein and marginally increased adenosine deaminase (ADA). Autoantibody testing was positive for antinuclear antibodies (ANA), but CSF culture and India ink staining were negative. Magnetic resonance imaging (MRI) of the brain revealed hyperintense lesions on T2-weighted and fluid-attenuated inversion recovery (FLAIR) images in the left peritrigonal and parietal white matter, suggesting demyelination. The diagnosis of PML was confirmed by a positive JC virus PCR result from the CSF. The patient was started on combination antiretroviral therapy (cART) and supportive measures to improve immune status. This case underscores the importance of considering PML in patients with new-onset neurological symptoms and immunosuppression.

## Introduction

A rare demyelinating infectious disease of the central nervous system, progressive multifocal leukoencephalopathy (PML) usually manifests in immunosuppressed patients who have had immunosuppressive medication treatment, hematologic malignancies, or acquired immunodeficiency syndrome (AIDS) [1]. The only polyomavirus known to cause nervous system disease, the dormant John Cunningham (JC) polyomavirus, reactivates to cause the disease [2]. It is an oral or respiratory icosahedral double-circular deoxyribonucleic acid (DNA) virus with a diameter of around 40 nm that often enters the body in childhood [3]. The tonsils are the site of infection most often [4], and the virus then multiplies in the tissues of the kidneys. While most patients still carry the dormant virus, the global population's seroprevalence of the virus is thought to range from 40% to 60% [3]. The JC polyomavirus tends to reorganize its molecular structure and execute a lytic infection of oligodendrocytes and astrocytes inside the central nervous system of immunocompromised persons [5], despite the fact that it does not pose a threat to the majority of immunocompetent individuals.

Clinical observations, imaging results, and polymerase chain reaction (PCR) analysis, which detects JC virus DNA in cerebral fluid, all contribute to the diagnosis of PML. The first stage in the diagnosing process is brain magnetic resonance imaging (MRI), where afflicted regions show fluid-attenuated inversion recovery (FLAIR) and look hypodense on T1 but hyperintense on T2. Usually located in the frontal lobes, PML is a monofocal condition that arises at the grey-white interface. The cortex is untouched, whereas subcortical U-fibers and deeper white matter are typically damaged [6,7]. This sets it apart from other opportunistic diseases that are frequently observed in human immunodeficiency virus (HIV) patients. For instance, lesions related to multiple sclerosis and cytomegalovirus (CMV) typically appear in the periventricular white matter [5]. When determining a PML diagnosis, identifying demyelination, more especially of the white matter, is crucial. While each affected person experiences PML differently, common noticeable findings include clumsiness and increasing weakening, along with changes in personality, speech, and vision [8]. When motor dysfunction, cognitive deficits, and visual problems occur together, PML is frequently suspected [4]. These alterations ultimately cause disabilities and even fatal situations. When it comes to confirming the diagnosis of PML, the cerebrospinal fluid (CSF) PCR has demonstrated the best sensitivity and specificity [7,9]. The diagnosis is further supported by observations such as CD4 lymphocyte counts of less than 200 cells/mm^3^. Here, we present the case of a newly diagnosed retroviral patient with atypical symptoms of PML.

## Case presentation

A 42-year-old male, a day wager by profession, presented to the hospital with complaints of slurring of speech and difficulty in articulation, along with tingling sensations in both feet persisting for three months, accompanied by difficulty in walking, leading to instances of slipping of footwear unnoticed and swaying while walking. Additionally, he experienced weakness in the distal upper limbs and difficulty naming objects and people for the past two months. Furthermore, he reported pain and dark discoloration of the left great toe for the last month, along with a significant weight loss of 10-12 kilograms over three or four months. The patient denied any known comorbidities. He gives a history of tobacco chewing and chronic alcohol consumption, which he claims to have quit. 

Upon admission, the patient was afebrile, with a blood pressure of 110/80 mmHg, a pulse rate of 84 beats per minute, and a saturation of 97% on room air. He was conscious and oriented to time, place, and person, although he struggled to name certain objects and repeat sentences fluently. 

Cranial nerve examination revealed no abnormalities, and systemic examination revealed normal tone and power in all four limbs, with absent reflexes in the bilateral biceps, triceps, supinator, and ankle, while flickering was present at the knee. Cerebellar examination showed instability with eyes open and swaying during walking, along with a wide-based gait. Coordination was impaired during finger-nose, finger-nose-finger, and knee-heel tests, and pendular knee jerk was absent. Vibration sense was diminished on the right upper and lower limbs compared to the left side, while pain and temperature sensations were intact.

Blood investigations were performed, which revealed a hemoglobin (Hb) level of 10.6gm/dl, a normal total leucocyte count of 6060/mm^3^, and maintained platelet counts. Additionally, serology tests were performed, which revealed enzyme-linked immunosorbent assay (ELISA) positivity for HIV-1 antibodies. D-Dimer was grossly elevated which was 2900.

The CSF examination showed a purely lymphocytic profile with elevated protein levels at 107.1 mg/dL. The CSF adenosine deaminase (ADA) was slightly increased at 7.4 U/L. An autoantibody profile was conducted to exclude vasculitis, revealing ANA positivity with a titer of 1:160 in a cytoplasmic pattern. CSF culture and India ink staining were negative. However, the CSF viral panel showed JC virus positivity via PCR. The patient's CD4 count was 171.

**Figure 1:**
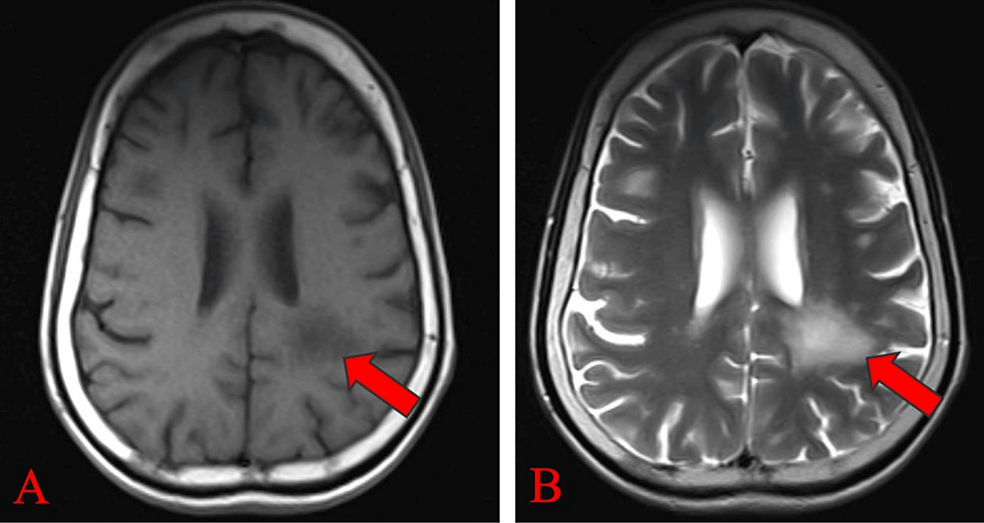
MRI brain (P+C) showing lesion in the left peritrigonal white matter extending in the left parietal white matter, which appears hypointense on T1-weighted image (A) and hyperintense on T2-weighted image (B), as shown by red arrows. MRI brain (P+C): Magnetic resonance imaging brain plain and contrast

**Figure 2:**
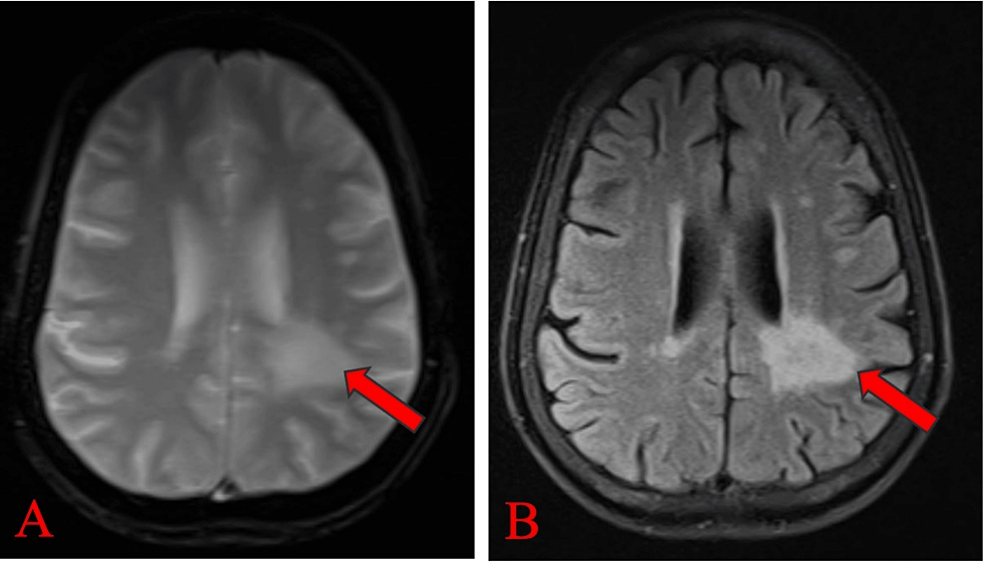
MRI brain (P+C) showing lesion in the left peritrigonal white matter extending in the left parietal white matter, which appears hyperintense on FLAIR (A), shows no blooming on GRE (B), as shown by red arrows. MRI brain (P+C): Magnetic resonance imaging brain plain and contrast; FLAIR: fluid-attenuated inversion recovery; GRE: gradient echo

**Figure 3:**
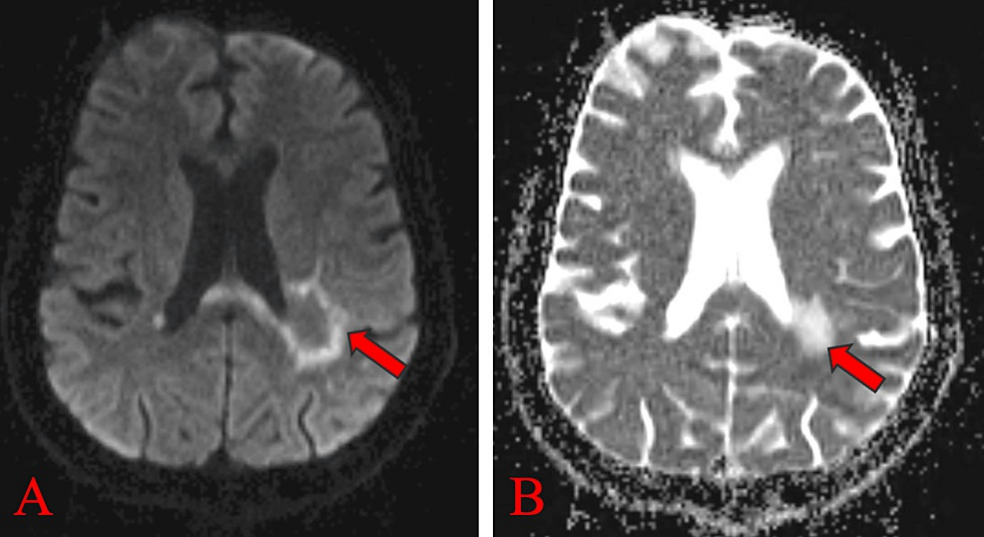
MRI brain (P+C) showing predominantly peripheral diffusion restriction on trace DWI image (A) with corresponding subtle hypointensity on ADC image (B) in the left peritrigonal region shown by red arrows. MRI brain (P+C): Magnetic resonance imaging brain plain and contrast; DWI: diffusion-weighted image; ADC: apparent diffusion coefficient

**Figure 4:**
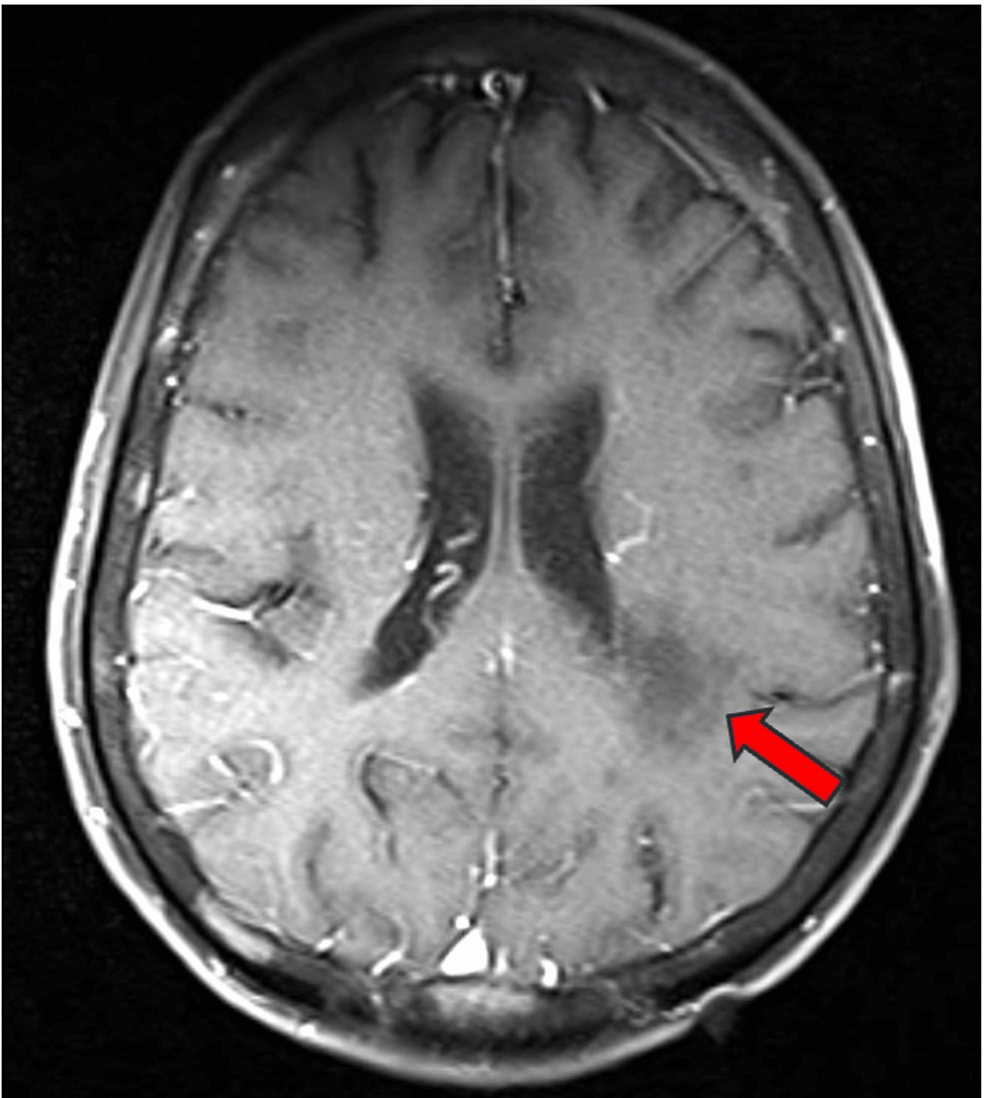
MRI brain (P+C) T1 post-contrast axial weighted image showing no enhancement of the lesion in the left peritrigonal region is shown by the red arrow. MRI brain (P+C): Magnetic resonance imaging brain plain and contrast

Magnetic resonance imaging (MRI) of the brain, both plain and contrast, revealed multiple hyperintense lesions in the left peritrigonal white matter extending into the left parietal white matter on T2-weighted images (Figure 1B) and FLAIR images (Figure 2A). These lesions appeared hypointense on T1-weighted images (Figure 1A). No blooming was observed on gradient echo (GRE) images (Figure 2B), while predominantly peripheral diffusion restriction was noted on trace diffusion weighted images (DWI) (Figure 3A), with corresponding peripheral subtle hypointensity on apparent diffusion coefficient (ADC) images (Figure 3B). The T1 post-contrast axial weighted images (Figure 4) showed no enhancement of the lesion in the left peritrigonal region. These findings suggest likely demyelination or leukoencephalopathy changes.

Based on these findings and the clinical examination, a differential diagnosis of PML was considered, particularly given the patients’ newly diagnosed retroviral status, along with possibilities of vasculitis or chronic infarcts. A working diagnosis of PML was established following the positive JC virus PCR result from the CSF analysis.

The patient was started on a combination antiretroviral therapy (cART) regimen consisting of tenofovir 300 mg, lamivudine 300 mg, and dolutegravir 50 mg once daily. Additionally, he received supportive treatments including azithromycin 500 mg once daily and trimethoprim (160 mg) + sulfamethoxazole (800 mg) once daily prophylactically to prevent further infections and enhance immune function.

At the two-month follow-up, the patient continued to experience challenges with word retrieval and concentration. Simple conversation or bedside examinations did not reveal any signs of aphasia. No signs of disease progression or immune reconstitution inflammatory syndrome were seen on neurological testing or repeated cerebral MRI.

The patient continued to experience stable, moderate issues with concentration and word retrieval at the seven-month follow-up, which were undetectable during conversation or neurological examination. The patient tolerated the treatment well, and no adverse events were reported.

## Discussion

Since only around 4000 diagnoses of PML are made annually in the combined United States and European Union, it can easily go undiagnosed or be misdiagnosed [10]. In India, PML in HIV/AIDS is uncommon. According to research by Sharma et al., advanced immunosuppression was prevalent at diagnosis, and the overall prognosis was dismal, with a median survival of 7.6 months in patients presented with PML [4]. When HIV-positive people still have sufficient CD4+ T cells in their bodies at the beginning of their illness, the JC virus finds it difficult to replicate. This can hinder early infection detection, even though it generally leads to better outcomes for PML patients [1].

Failure to diagnose PML might also stem from inconclusive diagnostic imaging data. The capacity to recognize clinical signs that may indicate an early presentation of PML is necessary in order to deliver timely and targeted therapeutic therapy. Findings such as punctate pattern, subcortical U-fiber involvement, and enhanced signal in diffusion-weighted imaging, in addition to T1 hypointensity [5], can be shown by MRI. Bilateral asymmetric nonenhancing multifocal regions of periventricular and subcortical white matter demyelination are significant additional differentiating criteria. When immune-compromised patients present with increasing neurologic symptoms and white matter lesions, there should be a strong clinical suspicion of PML.

Additionally, when acute diseases or those with a more obvious cause are given priority, PML may be disregarded and go undetected. All PML cases seen at Harvard Medical School's Neuro-Infectious Diseases Clinic between 1993 and 2015 were retrospectively reviewed. Among the 111 PML patients included, vascular pathologies (33%) and infectious pathologies other than PML (16%), as well as tumors (14%), were the most frequent initial diagnoses that led to additional work-up and delayed the diagnosis of PML [10]. For both HIV-positive and HIV-negative individuals, the median duration from the onset of symptoms to the diagnosis of PML was 74 days [10].

It is necessary to enhance screening protocols in order to detect asymptomatic PML infections and avoid misdiagnosing strokes. According to research from the University of Connecticut School of Medicine's Department of Neurology, just 27 (0.96%) of the 2806 patients who were diagnosed with ischemic strokes between January 2005 and May 2011 had HIV testing done [11]. An MRI of one study participant revealed lesions in the brain's left parietal and left thalamic areas. The patient also experienced stroke-like symptoms, such as weakness in the right hand's flexion and extension. Transesophageal echocardiography results were normal, suggesting that the patient may have suffered a cryptogenic stroke. Over the course of the following month, his symptoms worsened, and he ended up in the hospital as a result of his right-sided hemiparesis. An MRI revealed both new lesions in the right hemisphere and the progression of lesions in the left hemisphere. His immunocompromised state was suspected due to his CD4 level of 47 cells/uL. PML was confirmed by a subsequent positive PCR for the JC virus in the cerebral fluid. The patient died 4.5 months after the beginning of symptoms, and no antiretroviral medication was given due to aspiration risk [11]. By implementing screening programs for HIV infection, clinical suspicions of infection can be raised early, leading to earlier treatment and better results. A total of 50% of HIV-related PML treated with highly active antiretroviral therapy (HAART) survive for a year, compared to 10% of those who do not [1].

When treating immunocompromised individuals who experience recurrent, worsening neurologic symptoms, clinicians ought to seriously consider PML. Early detection in clinical practice can be enhanced by putting the Centers for Disease Control and Prevention (CDC) screening guidelines into practice. Patients with suspected chronic ischemic stroke may have worse patient outcomes, higher morbidity, and reduced HAART efficacy if they are not tested for HIV.

## Conclusions

This case emphasizes the importance of considering PML in the differential diagnosis of immunocompromised patients presenting with new-onset neurological symptoms. Our patient, a 42-year-old male recently diagnosed with HIV, exhibited a range of neurological deficits including slurred speech, coordination impairment, and sensory disturbances. The combination of clinical findings, characteristic MRI abnormalities, and the detection of JC virus DNA in the CSF through PCR confirmed the diagnosis of PML. Initiating HAART was essential to improve the patient's immune status and manage the infection.

This case illustrates the diagnostic challenges of PML, a condition that often mimics other neurological disorders. Early recognition and diagnosis are important for improving patient outcomes, particularly in HIV-positive individuals with advanced immunosuppression. The integration of clinical evaluation, advanced imaging techniques, and molecular diagnostics such as PCR for JC virus is pivotal in the accurate and timely diagnosis of PML. Due to the serious and potentially fatal nature of PML, it is crucial to have a high level of awareness and to promptly start HAART and supportive care in order to effectively manage this rare condition.
